# Anethole attenuates motor dysfunctions, striatal neuronal activity deficiency and blood brain barrier permeability by decreasing striatal α-synuclein and oxidative stress in rotenone-induced Parkinson’s disease of male rats

**DOI:** 10.1371/journal.pone.0294612

**Published:** 2023-11-16

**Authors:** Sadegh Moradi Vastegani, Seyed Esmaeil Khoshnam, Samireh Ghafouri, Nima Bakhtiari, Yaghoob Farbood, Alireza Sarkaki

**Affiliations:** 1 Persian Gulf Physiology Research Center, Medical Basic Sciences Research Institute, Ahvaz Jundishapur University of Medical Sciences, Ahvaz, Iran; 2 Department of Physiology, Medicine Faculty, Ahvaz Jundishapur University of Medical Sciences, Ahvaz, Iran; 3 Pain Research Center, Imam Khomeini Hospital, Ahvaz Jundishapur University of Medical Sciences, Ahvaz, Iran; 4 Medicinal Plant Research Center, Ahvaz Jundishapur University of Medical Sciences, Ahvaz, Iran; Federal University of Ceara: Universidade Federal do Ceara, BRAZIL

## Abstract

**Introduction:**

Anethole is the main compound of the essential oil of anise and several other plants, which has antioxidant, anti-inflammatory, and neuroprotective properties. Oxidative stress is considered as an important factor in the pathogenesis of PD. In the present study, we aimed to investigate the effects of anethole against rotenone-induced PD.

**Methods:**

Male Wistar rats were randomly divided into six groups. Control group received DMSO + sunflower oil, model group received rotenone (2 mg/kg, s.c, daily for 35 days), positive control group received L-Dopa, and test groups received anethole (62.5, 125, and 250 mg/kg, i.g, daily for 35 days) 1 hour before each rotenone injection. Body weight changes, rotarod test, stride length test, and extracellular single unit recording were performed after treatment. After behavioral test, Brain water content and blood brain barrier (BBB) permeability were evaluated, and the levels of malondialdehyde (MDA), superoxide dismutases (SOD), alpha-synuclein and MAO-B were measured in the striatum.

**Results:**

Chronic administration of rotenone induced body weight loss and caused significant dysfunction in locomotor activity, neuronl firing rate, and BBB. Rotenone also decreased SOD activity, increased MDA level, and elevated the expression of alpha-synuclein and MAO-B in the striatum. However, treatment with anethole attenuated body weight loss, motor function, neuronal activity, and BBB function. Furthermore, Anethole treatment attenuated oxidative stress and decreased the expression of alpha-synuclein and MAO-B compared to the rotenone group.

**Conclusion:**

Our results show that through its antioxidant properties, aethole can improve the cellular, molecular and behavioral characteristics of rotenone-induced Parkinson’s disease.

## 1. Introduction

Parkinson’s disease (PD) is a highly prevalent neurodegenerative disease characterized by degeneration of dopaminergic neurons in the substantia nigra pars compacta (SNpc) and intracytoplasmic accumulation of Alpha-synuclein (α-syn) in insoluble structures called Lewy bodies (LBs) [1]. The clinical features of PD include bradykinesia, akinesia, muscle rigidity, resting tremor, and postural instability [2]. However, most patients with PD show at least one non-motor symptoms (NMS) such as fatigue, insomnia, anxiety, depression, and cognitive dysfunction [3].

Pesticides have been widely used by various routes of administration to induce pathological and clinical characteristics of PD in animal models [4, 5]. Rotenone, as a pesticide and inhibitor of mitochondrial complex I, has been widely used by a rout of systemic administration to produce experimental models of PD in rodents [6, 7]. Due to its lipophilic properties, rotenone easily passes through the BBB, which can be a reason for its neurotoxicity after systemic injection [8]. Studies have shown that systemic administration of rotenone induces α-syn accumulation and degeneration of dopaminergic neurons, resulting in PD-like symptoms including motor and non-motor disorders [9]. Studies have also demonstrated that rotenone exposure causes dopaminergic neuronal apoptosis and synaptic loss through microglia-mediated BBB dysfunction [10]. Rotenone reduces ATP production and increase reactive oxygen species (ROS) by inhibiting the mitochondrial electron transport chain (ETC) complex I, which can induce oxidative stress [11]. Therefore, antioxidant compounds can be effective in the prevention and treatment of PD by limiting the production and over accumulation of ROS.

Anethole (1-methoxy-4-benzene-[1-propenyl]) is an aromatic compound and is the main constituent (90%) of star-anise essential oil, which is widely used in the food industry. Studies demonstrated that anethole has anti-inflammatory [12], antioxidant [13, 14], antifungal [15], antibacterial [16], analgesic [17], and anesthetic [18] effects. Furthermore, anethole has been shown to exerts anti-inflammatory and neuroprotective effects against neurodegenerative diseases [17, 19]. Anethole significantly ameliorates neuronal cell death in an in vitro model of ischemia through its antioxidant and anti-excitotoxic effects as well as mitochondrial protection [20]. The beneficial effects of anethole in neurological disorders may also be due to its inhibitory properties on acetylcholinesterase (AChE) [21]. In previous studies, we demonstrated that aethole has neuroprotective effects against rotenone-induced motor and non-motor disorders such as depression, anxiety, and memory deficits in a rat model of PD [22, 23]. In addition, it has also been shown that Anetholedithiolethione (a synthetic analogue of anethole) plays an important role in the development of neuroprotective agents in PD, through its antioxidant activity as well as inhibition of monoamine oxidase (MAO)-B activity [24]. The MAO-degraded oxidation of dopamine produces oxidative stress that is implicated in dopaminergic neuronal degeneration in the substantia nigra, a relevant phenomenon in the pathogenesis of PD [25]. Therefore, anethole might be a good therapeutic candidate for neurological diseases such as Alzheimer’s diseases (AD) and PD.

As mentioned above, anethole has antioxidant and anti-inflammatory effects on the nervous system, but its molecular mechanisms on PD and related movement disorders have not yet been extensively investigated.Therefore, in this study, we investigated the effects of anethole in rotenone-induced PD via systemic (subcutaneous; s.c.) administration as an experimental nutritional poisoning model in Rats.

## 2. Materials and methods

### 2.1. Agents

Rotenone and Anethole were purchased from Sigma Chemical Company (St. Louis, MO, USA). Dimethyl sulfoxide (DMSO) was obtained from Merck Company (Darmstadt, Germany). All chemicals and reagents were of analytical grade. Anti-α-syn and anti-MAO-B were purchased from Santa Cruz Biotechnology, Inc., USA.

### 2.2. Animals

Eight-week-old male Wistar rats (200–220 g) purchased from Ahvaz Jundishapur University of Medical Sciences animal care and breeding center, Ahvaz, Iran. Animals were housed individually in standard Plexiglas cages under controlled conditions of temperature (22 ± 2°C) and 12 h light/dark cycle (lights on at 7 a.m.), with food and water *ad libitum*. All experiments were performed in Ph.D. thesis project according to the guidelines established by the National Institutes of Health (NIH) for the care and use of laboratory animals and approved by the Institutional Animal Ethics Committee of Ahvaz Jundishapur University of Medical Sciences (Ethics code: IRAJUMS.ABHC.REC.1400-053).

### 2.3. Experimental design

The animals were divided into the following groups:

Group I: Control; rats received DMSO + sunflower oil (1 ml/kg, s.c.), once daily for 35 days.

Group II: Rotenone (Rot); rats received rotenone (2 mg/kg, s.c.), once daily for 35 days.

Group III: Rot + Ant_**62.5**_; rats received anethole (62.5 mg/kg, i.g.) and rotenone (2 mg/kg, s.c.), once daily for 35 days.

Group IV: Rot + Ant_**125**_; rats received anethole (125 mg/kg, i.g) and rotenone (2 mg/kg, s.c.), once daily for 35 days.

Group V: Rot + Ant_**250**_; rats received anethole (250 mg/kg, i.g.) and rotenone (2 mg/kg, s.c.), once daily for 35 days.

Group VI: Rot + L-DOPA: PD rats received mixture of L-DOPA/Carbidopa (100 mg/kg, 10 mg/kg, i.p. respectively) 1h prior to the rotenone administration (2 mg/kg, s.c), once daily for 35 days.

**Fig 1:**
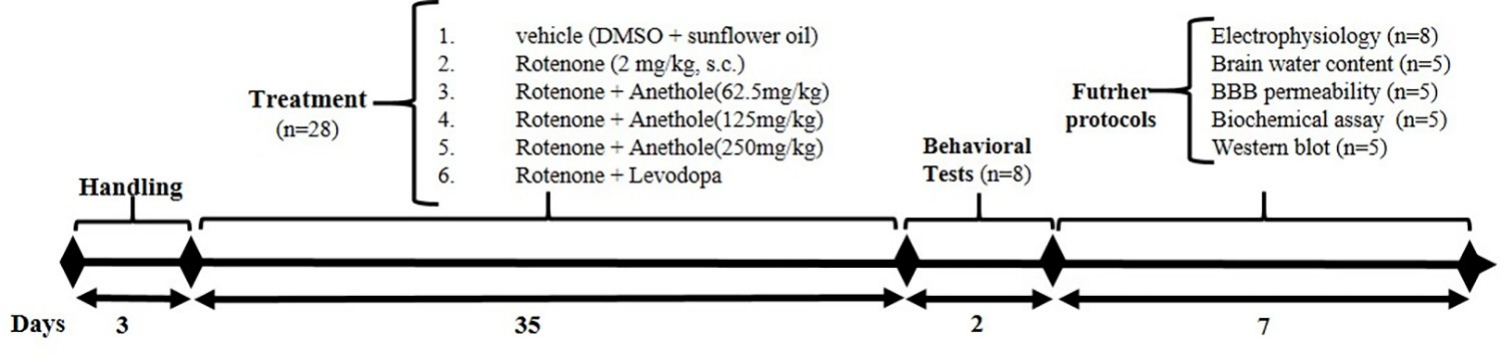
Schematic representation of time line and experimental protocols.

Rotenone was first dissolved in DMSO and then diluted in sunflower oil to obtain a final concentration of 2 mg/kg rotenone in 2% DMSO, 98% sunflower oil. Sunflower oil with 2% DMSO was used as the control. Anethole was dissolved in distilled water and administered by intragastric (i.g.) route 1h prior to the rotenone administration. Time line and design of experimental protocols in current work was shown in Fig 1.

### 2.4. Behavioral and non-behavioral tests

#### 2.4.1. Body weight measurement

Animal’s body weight was measured one day before rotenone administration (first day) and on the last day of the administration (35^th^ day). The percentage of weight change was calculated by using the following equation [26]: -[Weight changes (1^st^ day-35^th^ day)/1^st^ day body weight] * 100.

#### 2.4.2. Motor coordination test

Rotarod device was used to evaluate motor coordination (balance) in experimental rodents. Rats were acclimated to the apparatus 24 h before starting the tests. After 24 h rats were placed again on a turning rod accelerating from 4 to 40 rotations per minute (rpm) in 5 minutes. The latency to fall from the turning rod was recorded for each animal. This experiment was repeated three times on the day of testing with 45 min intervals [27].

#### 2.4.3. Stride length test

The stride-length apparatus was used to examine stepping patterns in animals. The homemade woody apparatus consisted of a walkway section (40 cm long x 5 cm wide x 10 cm high) connected to a dark wooden box (20×20×10 cm). The rats’ forepaws were inked and they were then allowed to walk over a paper strip in the walkway section toward the dark box to make a footprint. Stride length was determined as the distance (in mm) between two consecutive footprints. Four step lengths were measured from each animal for statistical analysis [28].

### 2.5. Electrophysiological recording

Parylene-coated tungsten microelectrodes (with sharp tip of ~1 μm and impedance of ~10 MΩ; WPI, Sarasota, FL, USA) were used to investigate the firing activity of striatal neurons in rats as extracellular single unit recording. After anesthesia with urethane (1.5 g/kg, i.p.), the animals were fixed in a stereotaxic frame and then a 2-mm diameter hole was created in the right part of the skull above the striatum (from bregma: AP  =  + 0.5 mm; ML  = ± 2.8 mm; according to the rat brain atlas of Paxinos & Watson) by minidrill. To record the best neuronal spikes, the microelectrode was brought down to the striatum by a micromanipulator (from skull surface: DV ~ 5.5 mm). The spike signals received by the electrode were amplified (gain 1000) and filtered (band-pass 0.3–10 kHz), and sampled at 50 kHz using a Neurolog system (D3109; WSI, Tehran, Iran). In addition, a window discriminator (W3205; WSI, Tehran, Iran) was used to identify and classify spikes based on their amplitude. The frequency of spikes was counted (in time bins of 1000 ms) and shown by online-sorter software (Spike; Science Beam, Tehran, Iran) [29].

### 2.6. Brain water content

After behavioral tests, rats (n = 5) were anesthetized deeply and irreversibly by an overdose sodium thiopental (Nesdonal, 100 mg/kg) and then decapitated. Whole rat brains were extracted and weighed immediately as wet weight. Afterward, we put the brains in an oven at 100°C for two days and then weighed again as dry weight. The brain water content (BWC) was calculated by using the following equation [30]: BWC = [(wet weight–dry weight)/ (wet weight)] ×100%.

### 2.7. Evaluation of blood-brain barrier (BBB) permeability

We used Evans blue (EB) staining to evaluate the BBB permeability. Under deep anesthesia with an overdose sodium thiopental, 2% EB solution in normal saline was slowly injected through the tail vein in rats (5 mL/kg). Two hours later, rats were perfused with saline through the left ventricle to washout residual dye in the intracerebral blood vessels. After decapitation, the brain samples were collected and cerebral hemispheres were isolated and weighed separately. The samples were homogenized in 1.5 ml of PBS and centrifuged at 15,000 ×g m for 20 min. The supernatant was collected and mixed with an equal amount of trichloroacetic acid (TCA), and then incubated for 24 hours at 4°C. The mixture was centrifuged again (12,000 x g for 20 min) and the supernatant was collected. Finally, the absorbance of supernatant was quantified with a spectrophotometer at a wavelength of 620 nm. The concentration of EB dye in the brain tissue were quantified using a standard curve of EB in the same solvent and were presented as μg/g of brain tissue [31].

### 2.8. Biochemical assay

After behavioral tests, five animals in each group were deeply and irreversibly anesthetized by an overdose sodium thiopental (Nesdonal, 100 mg/kg, i.p.). Immediately after decapitation, the brains were carefully removed from the skull, and the striatum was separated on ice. The samples were stored at -80°C until further analysis. In order to analysis the biochemical parameters, the striatal tissue was weighed and homogenized with 0.1 M phosphate buffer (pH 7.4). The obtained homogenate was centrifuged for 10 min at 3000 r/min at 4°C and the supernatant was separated for analysis. The concentration of protein was determined in the supernatant by the Bradford method [32].

#### 2.8.1. Estimation of lipid peroxidation

Chromatographic determination of malondialdehyde (MDA) was measured to observe lipid peroxidation. In brief, the tissue samples (50 μL) were incubated in a boiling water bath for 20 min in the presence of 150 μL of 20% Trichloroacetic acid (TCA) and 100 μL of 6% Thiobarbituric acid (TBA). The dilution was then cooled on ice and centrifuged at 5000 rpm for 5 min at 4°C to remove impurities. Finally 200 μL of the clear supernatant was transferred to a microplate and the absorbance was measured at 535 nm using a spectrophotometer (Bio-Tek, Winooski, VT, USA). The concentrations of MDA were determined using a standard curve and results were expressed as μM/mg protein [33].

#### 2.8.2. Evaluation of superoxide dismutase (SOD) activity

The activity of SOD was measured according to the method of Kono et al. [34]https://www.ijnpnd.com/article.asp?issn=2231-0738;year=2018;volume=8;issue=2;spage=53;epage=58;aulast=Balakrishnan - ref16. Briefly, in this method hydroxylamine hydrochloride (NH2OH.HCl) was added to the reaction mixture containing nitroblue tetrazolium (NBT) dye to generate the superoxide anion. NBT is reduced by superoxide anion to formazon, but SOD removes this anion and so inhibits the reduction of NBT. The inhibitory effect of SOD on NBT reduction was measured as SOD activity. The activity of SOD was expressed as U/mg proteins.

### 2.9. Western blotting

Western blot analysis was carried out to examine the expression levels of a-syn and Monoamine oxidase-B (MAO-B) in the rat striatum. The striatum samples were homogenized in RIPA lysis buffer (50 mM Tris, 150 mM NaCl, 0.1% SDS, 1% sodium deoxycholate, 1% Triton X-100) in the presence of a protease inhibitor and centrifuged for 15 min at 10,000×g at 4°C. Protein concentrations were calculated using the Bradford protein assay. Samples containing 50 μg total protein were loaded and separated through12% SDS-PAGE gel and subsequently transferred to polyvinylidene difluoride (PVDF) membranes by electrophoresis. The PVDF membranes were blocked with Tris-buffered saline plus 0.1% Tween 20 (TBST) containing 5% skim milk powder for2 hours at room temperature. After blocking, the membranes were probed overnight at 4°C with the following primary antibodies: anti-α-syn (sc-12767, Santa Cruz Biotechnology) and anti-MAO-B (sc-515354, Santa Cruz Biotechnology). The next day, the PVDF membranes were washed 3 times with TBST and then incubated with a horseradish peroxidase (HRP)-conjugated secondary antibody for 2 hour at room temperature. The PVDF membranes were washed again with TBST and the protein bands were visualized using enhanced chemiluminescence (ECL) reagents in Medical Automatic X-ray Film Processor (LD-14; China). The bands were then scanned and quantified by JS-2000 Scanner (BonninTech; China) [35].

### 2.10. Statistical analysis

Statistical analysis was performed using GraphPad Prism version 8.00 (GraphPad Software, La Jolla California USA, www.graph.pad.com). The results were expressed as mean ± standard error of the mean (S.E.M) and were analyzed by one-way analysis of variance (ANOVA), followed by Tukey’s honestly significant difference post-hoc test. Statistical significance was set at P < 0.05.

## 3. Results

### 3.1. Body weight

**Fig 2:**
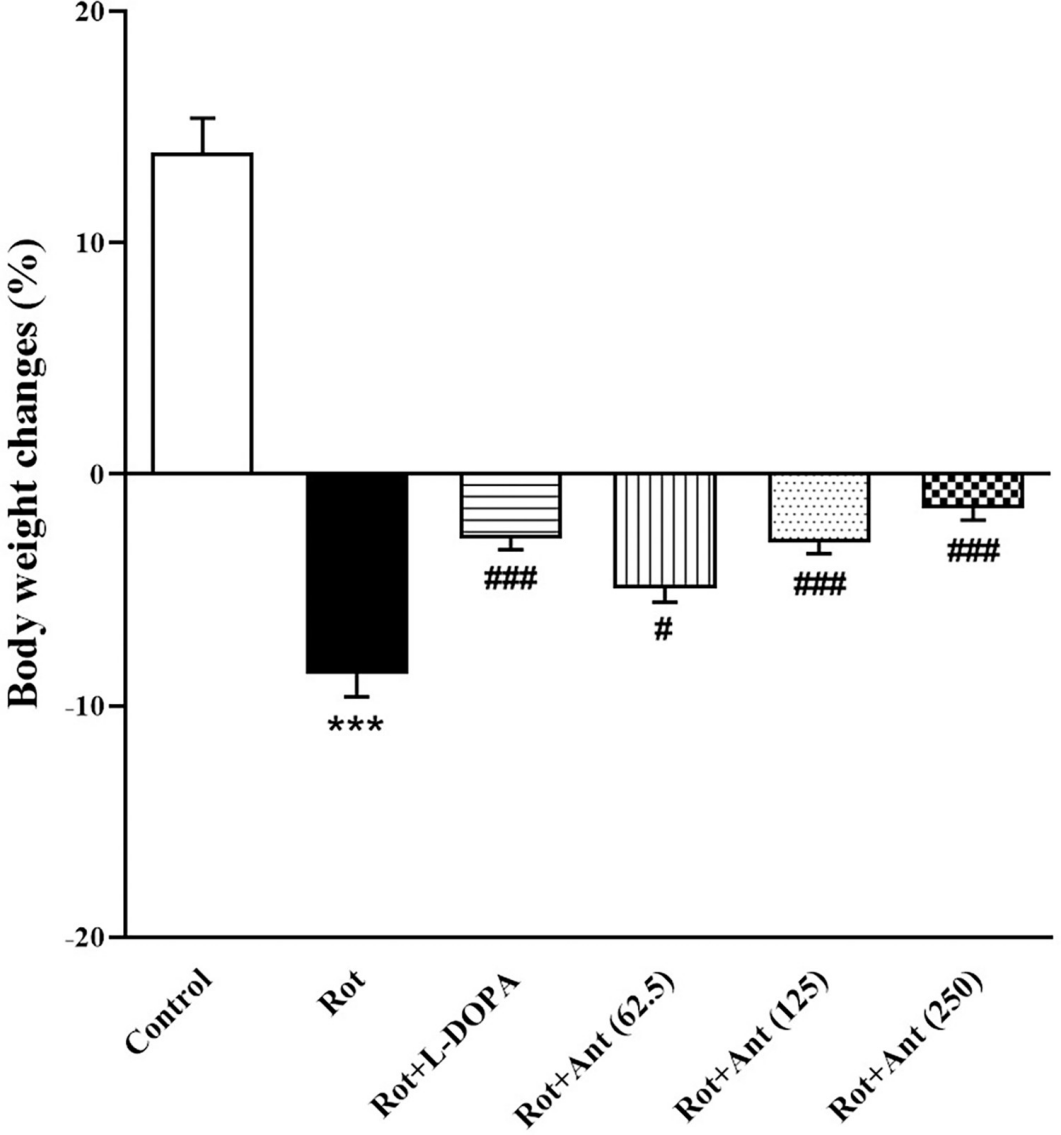
Effect of anethole on body weight changes in all tested groups. Data are presented as the mean ± S.E.M (n = 10). Significant differences were determined using one-way ANOVA followed by Tukey’s test. ***P < 0.001 vs. the control group; ^#^P < 0.05, ^###^P < 0.001 vs. the rotenone group. (Rot = rotenone; L-DOPA = levodopa; Ant = anethole).

Rotenone treated rats demonstrated a significant body weight loss during the period of administration compared with the control group (p < 0.001). But treatment with L-Dopa significantly attenuated the body weight (p < 0.001) compared with the Rotenone-treated group. In addition, anethole (62.5, 125, and 250 mg/kg) treatment significantly attenuated body weight loss (p < 0.05, p < 0.001, and p < 0.001 respectively) compared with the rotenone group. However, body weight in the L-Dopa, anethole 62.5, anethole 125, and anethole 250 groups were still lower than the control group (p < 0.001, p < 0.001, p < 0.001, and p < 0.001 respectively) (Fig 2).

### 3.2. Rotarod test

**Fig 3:**
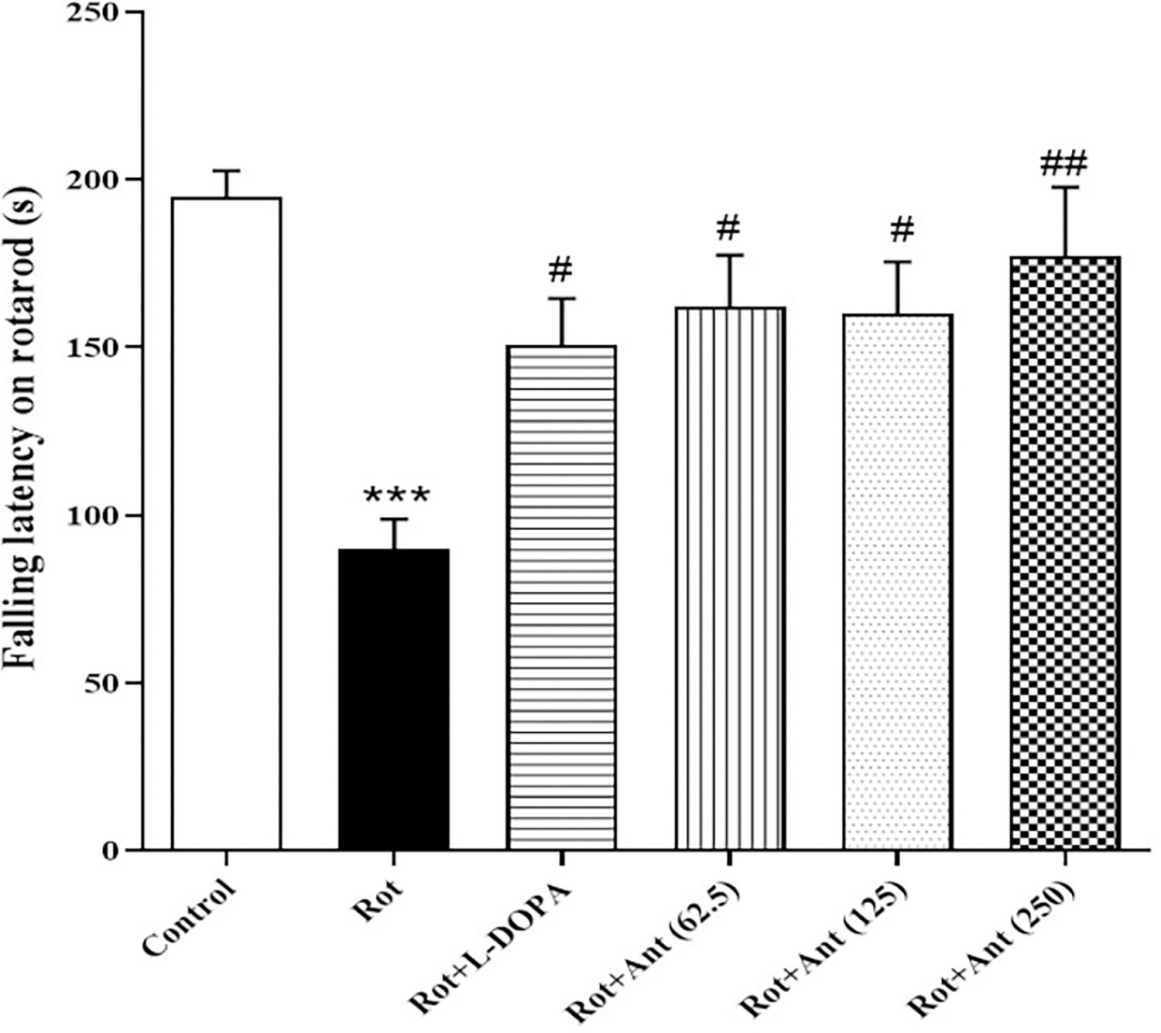
Effect of anethole on motor coordination in all tested groups. Data are presented as the mean ± S.E.M (n = 8). Significant differences were determined using one-way ANOVA followed by Tukey’s test. ***P < 0.001 vs. the control group; ^#^P < 0.05, ^##^P < 0.01 vs. the rotenone group. (Rot = rotenone; L-DOPA = levodopa; Ant = anethole).

As shown in Fig 3, rotenone impaired the motor coordination and in the rotarod test the falling latency was significantly shorter in rotenone group compared with the control group (p < 0.001). However, treatment with L-Dopa significantly increased the falling latency of PD rats (p < 0.05). In addition, anethole (62.5, 125, and 250 mg/kg) treatment significantly increased the falling latency in the rotenone-treated rats (p < 0.05, p<0.05, and p<0.001 respectively).

### 3.3. Stride length

**Fig 4:**
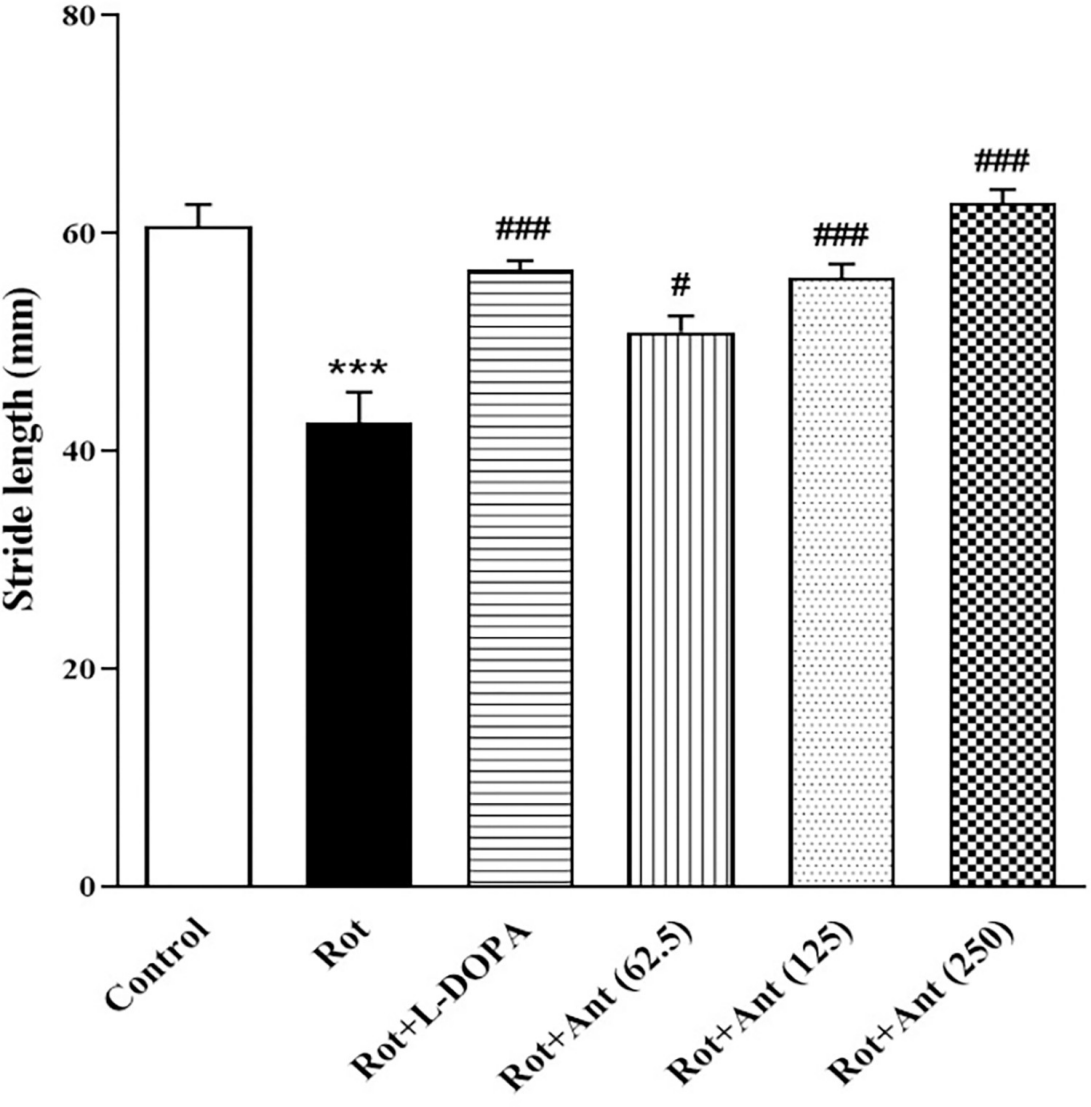
Effect of anethole on stride length in all tested groups. Data are presented as the mean ± S.E.M (n = 8). Significant differences were determined using one-way ANOVA followed by Tukey’s test. ***P < 0.001 vs. the control group; ^#^P < 0.05, ^###^P < 0.001 vs. the rotenone group. (Rot = rotenone; L-DOPA = levodopa; Ant = anethole).

As shown in Fig 4, stride length was significantly shorter in rotenone-treated rats compared with the control group (p < 0.001). Compared with the rotenone group, L-Dopa markedly increased the stride length of rats (p < 0.001). Anethole (62.5, 125, and 250 mg/kg) treated animals exhibited a significant increase in stride length compared with the rotenone group (p < 0.05, p<0.001, and p<0.001 respectively).

### 3.4. Neuronal firing rate in the striatum

**Fig 5:**
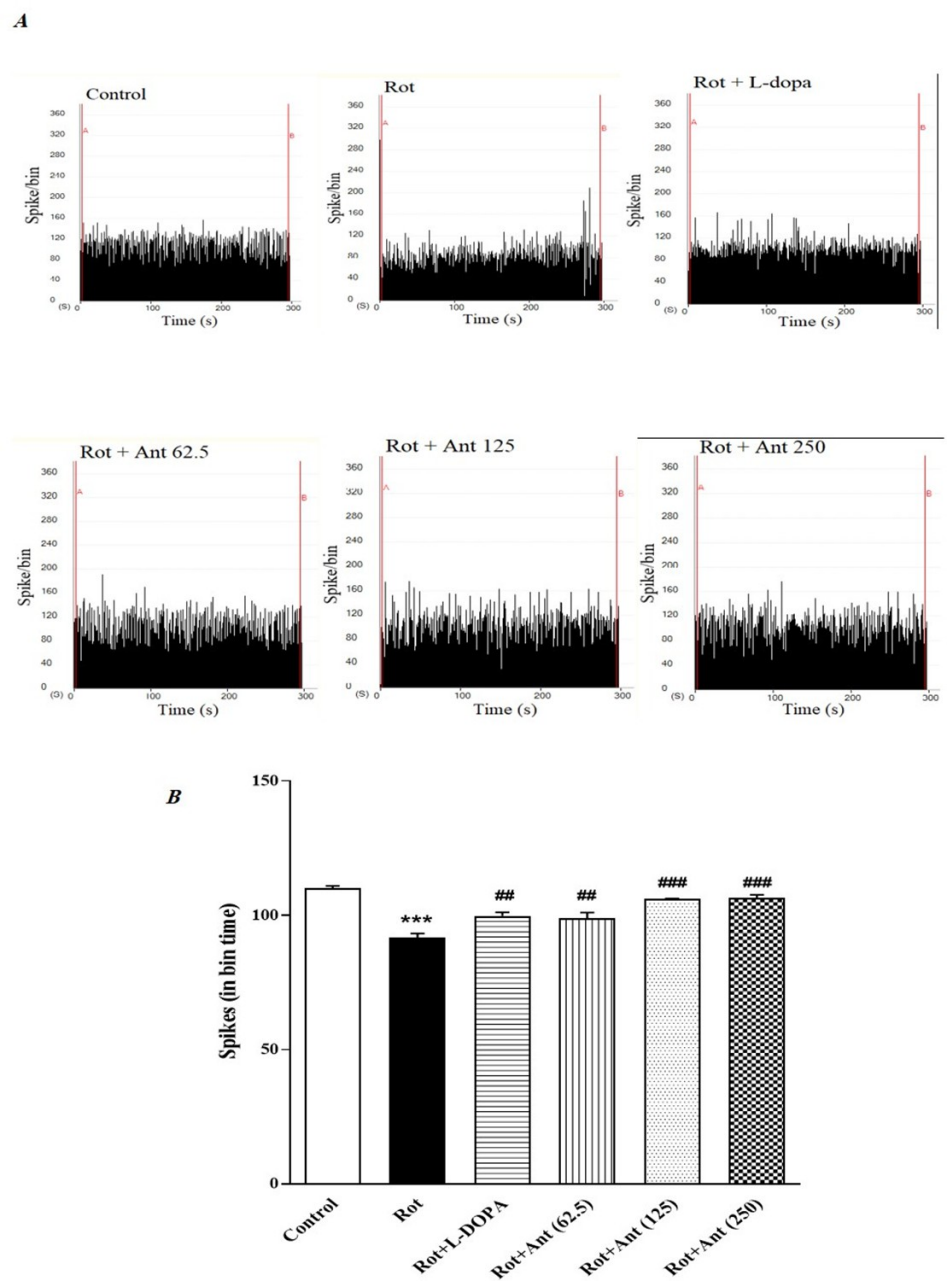
**A.** The trace of neuronal firing rate of striatum in different groups. **B.** Effect of anethole on average number of spikes/bin in rotenone treated rats. Data are presented as the mean ± S.E.M (n = 8). Significant differences were determined using one-way ANOVA followed by Tukey’s test. ***P < 0.001 vs. the control group; ^##^P < 0.01, ^###^P < 0.001 vs. the rotenone group. (Rot = rotenone; L-DOPA = levodopa; Ant = anethole).

To evaluate the effects of anethole on neuronal firing rate in the rat striatum, we used a single-unit recording technique. Representative traces of neuronal activity in the striatum are shown in Fig 5A. The numbers of spikes in 200 msec bin times were measured over 1200 seconds. The electrophysiological evaluation showed that rotenone administration significantly decreased the number of spikes/bin compared with the control group (p < 0.001). While the number of spikes/bin was significantly increased in L-Dopa treated rats in comparison to the rotenone group (p < 0.01). Furthermore, anethole (62.5, 125, and 250 mg/kg) treatment significantly and dose-dependently increased the spike rate related to rotenone group (p < 0.01, p<0.001, and p<0.001 respectively) (Fig 5B).

### 3.5. Brain water content

**Fig 6:**
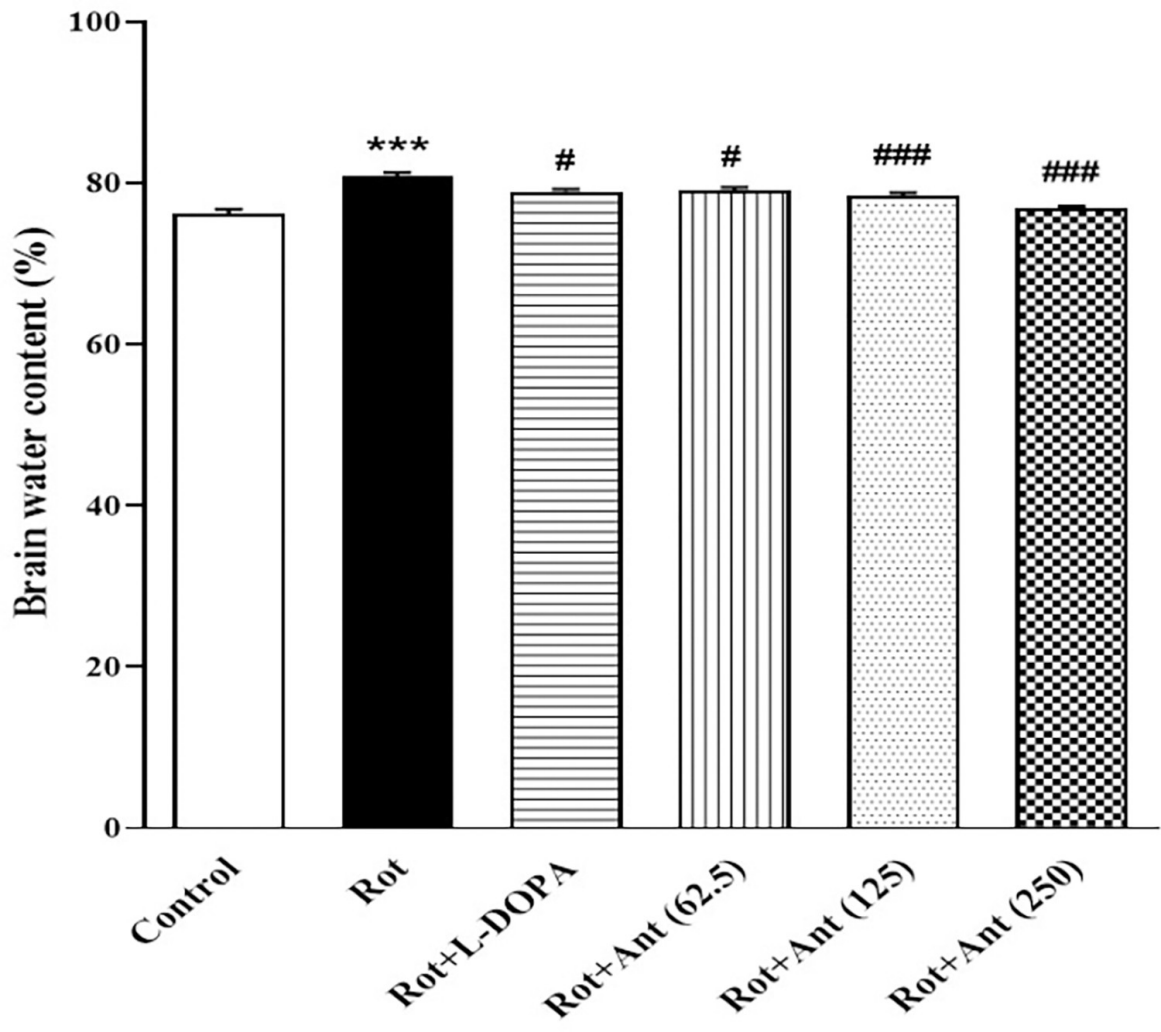
Effect of anethole on Brain water content in different tested groups. Data are presented as the mean ± S.E.M (n = 5). Significant differences were determined using one-way ANOVA followed by Tukey’s test. ***P < 0.001 vs. the control group; ^#^P < 0.05, ^###^P < 0.001 vs. the rotenone group. (Rot = rotenone; L-DOPA = levodopa; Ant = anethole).

Brain water content was significantly increased in rotenone-treated rats in comparison with control group (p < 0.001). Compared with the rotenone group, the brain water content significantly decreased in the L-Dopa treated group (p < 0.05). In rats treated with anethole (62.5, 125, and 250 mg/kg), significant increase of the brain water content was observed compared with the rotenone group (p < 0.05, p<0.001, and p<0.001 respectively) (Fig 6).

### 3.6. BBB permeability

**Fig 7:**
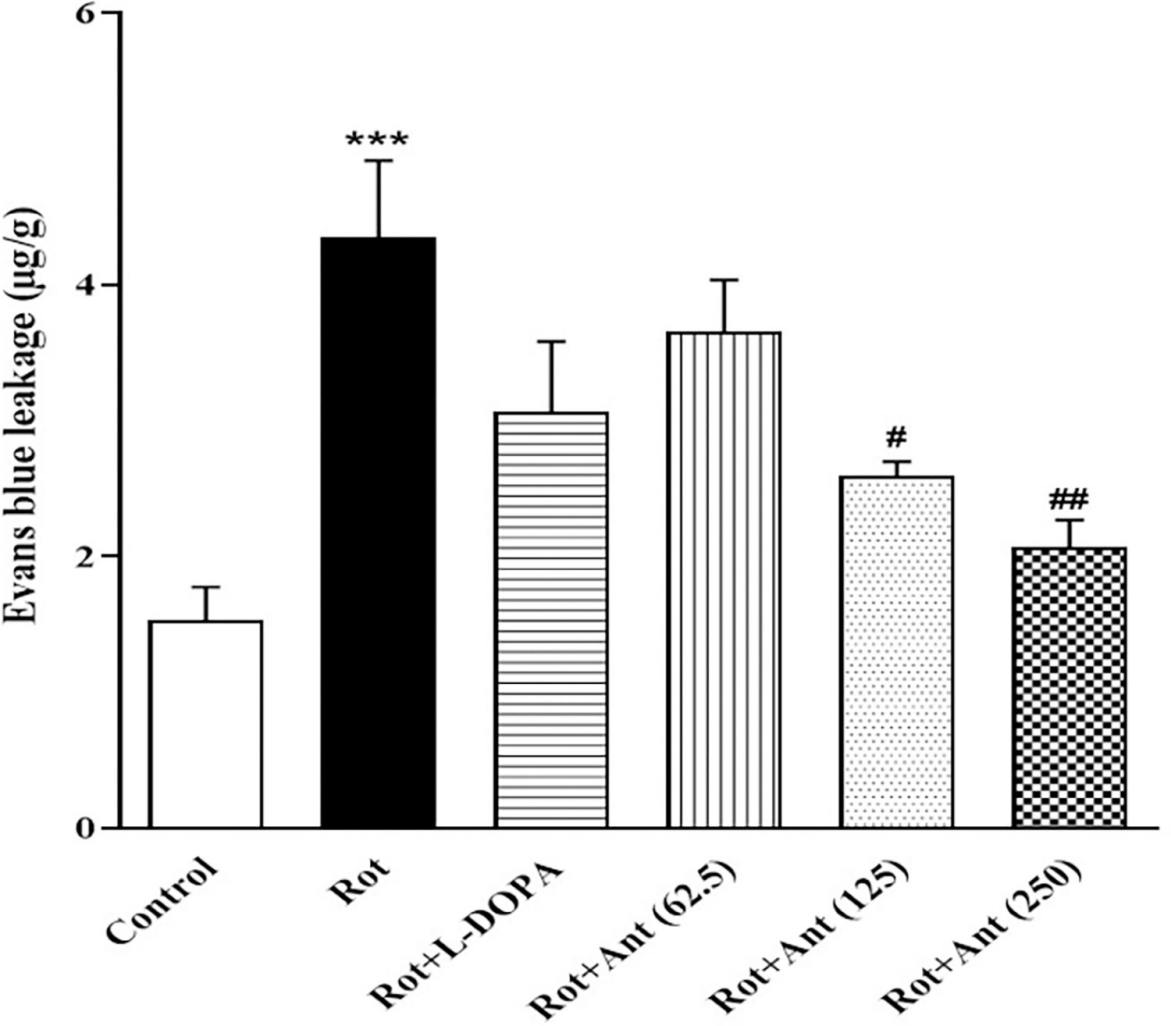
Effect of anethole on Evans blue content in all tested groups. Data are presented as the mean ± S.E.M (n = 5). Significant differences were determined using one-way ANOVA followed by Tukey’s test. ***P < 0.001 vs. the control group; ^#^P < 0.05, ^##^P < 0.01 vs. the rotenone group. (Rot = rotenone; L-DOPA = levodopa; Ant = anethole).

Evans blue content in the brain was measured to assess the BBB permeability in animals. Rotenone-treated rats had a significantly increased Evans blue content in comparison to the control group (p < 0.001). Administration of L-Dopa and low dose of anethole (62.5 mg/kg) did not change the Evans blue content in rats as compared to rotenone treated groups (p>0.05), while high doses of anethole (125 and 250 mg/kg) significantly decreased Evans blue content in rats’ brain in comparison with rotenone group (p<0.05, and p<0.01 respectively) (Fig 7).

### 3.7. Oxidative stress

**Fig 8:**
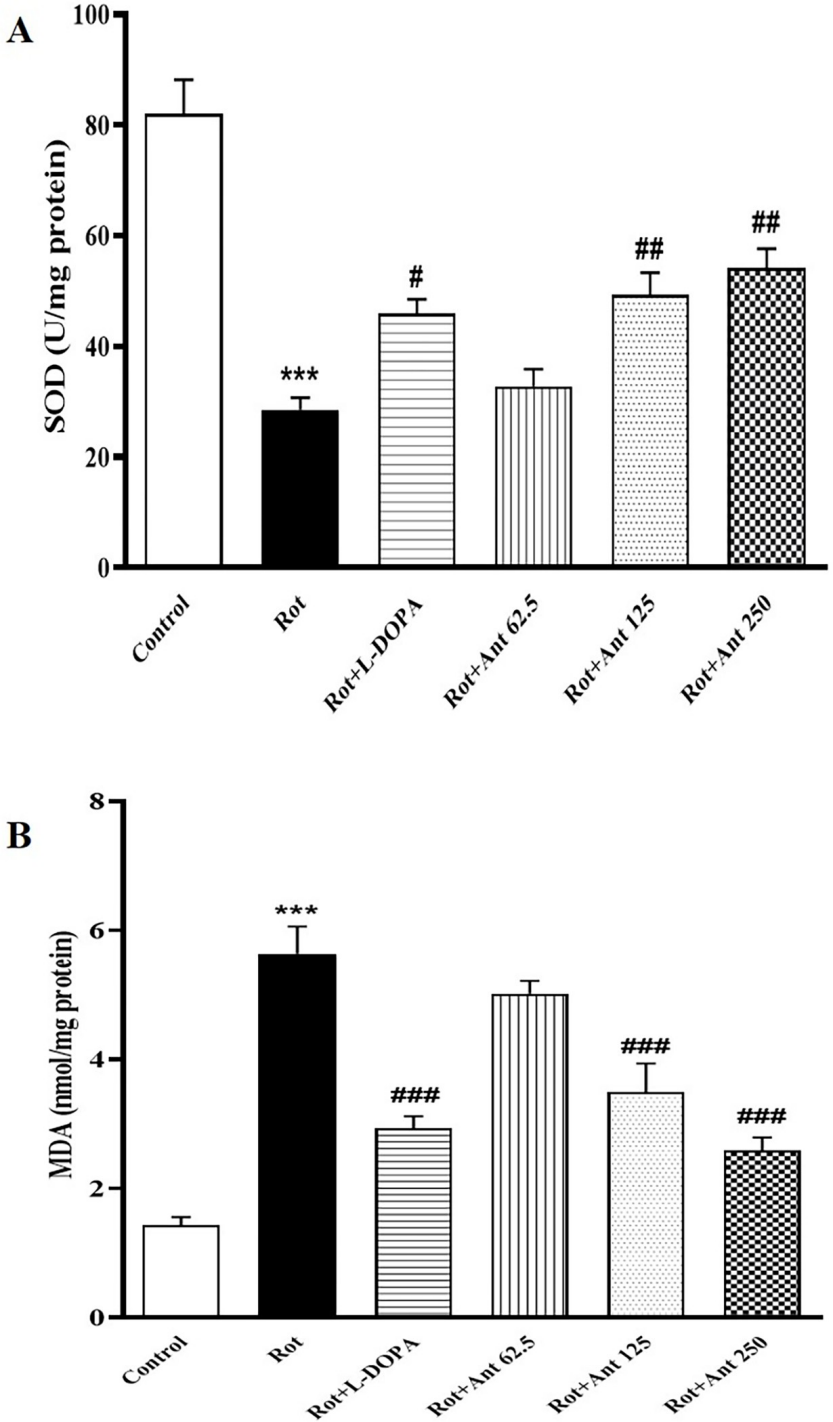
Effect of anethole on oxidative stress in all tested groups. Effects of L-Dopa and anethole on **(A)** SOD and **(B)** MDA activities in rats treated with rotenone. Data are presented as the mean ± S.E.M (n = 5). Significant differences were determined using one-way ANOVA followed by Tukey’s test. ***P < 0.001 vs. the control group; ^#^P < 0.05, ^###^P < 0.01, ^###^P < 0.001 vs. the rotenone group. (Rot = rotenone; L-DOPA = levodopa; Ant = anethole).

To investigate the effects of anethole on rotenone-induced oxidative stress in the rat brain striatum, MDA and SOD were evaluated using ELISA kits. SOD content was found to be decreased and MDA was increased significantly in rotenone treated rats in comparison to the control group (p < 0.001 and p<0.001 respectively). L-Dopa treatment significantly increased the levels of SOD and reduced the levels of MDA as compared to rotenone (p < 0.05 and p<0.001 respectively). Anethole at a low dose (62.5 mg/kg) did not change the levels of SOD and MDA (p>0.05). While high doses of anethole (125 and 250 mg/kg) significantly increased SOD activity (p < 0.01 and p<0.01 respectively) and decreased MDA levels (p < 0.001 and p<0.001 respectively) in rotenone-induced PD rats (Fig 8).

### 3.8. α-syn and monoamine oxidase-B (MAO-B) protein levels

**Fig 9:**
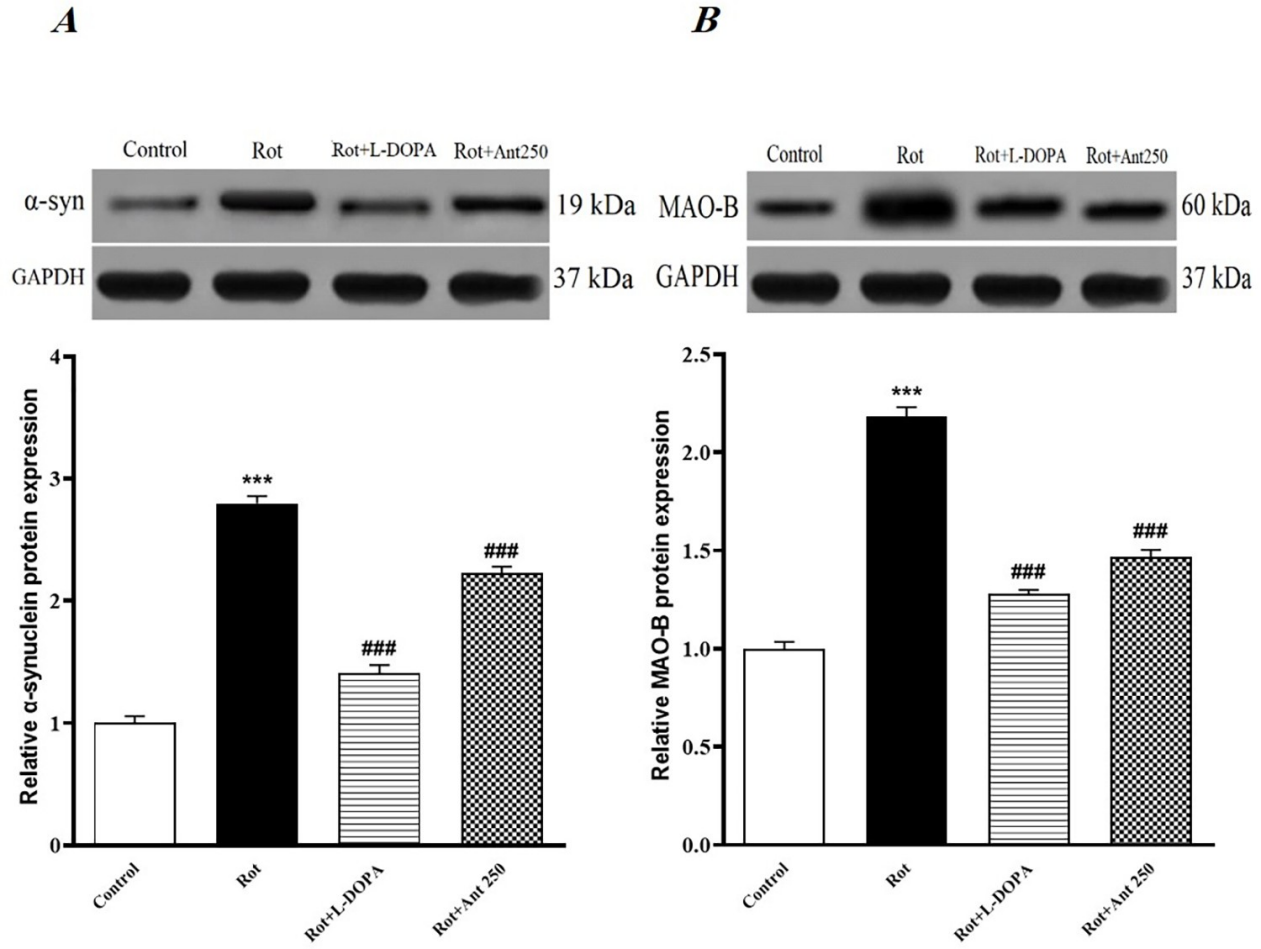
**A:** Represents the Western blot band images and the relative protein expression of α-syn (GAPDH served as the internal control). **B:** Represents the Western blot band images and the relative protein expression of MAO-B (GAPDH served as the internal control). Data are presented as the mean ± S.E.M (n = 5). Significant differences were determined using one-way ANOVA followed by Tukey’s test. ***P < 0.001 vs. the control group; ^###^P < 0.001 vs. the rotenone group. (Rot = rotenone; L-DOPA = levodopa; Ant = anethole).

The results of a western blot assay revealed that rotenone significantly up-regulated the protein levels of α-syn and MAO-B in comparison to the control group (p < 0.001 and p<0.001 respectively). While the protein levels of α-syn and MAO-B were significantly suppressed in L-Dopa treated rats compared with the rotenone group (p < 0.001 and p<0.001 respectively). In addition, treatment with anethole (250 mg/kg) significantly suppressed the protein levels of α-syn (p < 0.001 and p<0.001respectively) and MAO-B (p < 0.001 and p<0.001 respectively) in rotenone-induced PD rats (Fig 9).

### 4. Discussion

In this study, we evaluated the antioxidant and neuroprotective effects of anethole in a rat model of PD induced by rotenone. The results indicate that chronic administration of anethole attenuated body weight loss and motor deficits, and improved BBB dysfunction induced by rotenone. Moreover, we showed that rotenone-induced oxidative stress was attenuated by anethole administration. Additionally, we demonstrated that anethole decreased the protein expression of α-syn and MAO-B in the striatum of rotenone- lesioned rats. Our findings may provide new support for anethole as a therapeutic strategy for PD.

Various strategies for the prevention or treatment of PD have been widely investigated in recent years [36]. Oxidative stress has gained attention as one of the main processes responsible for the pathogenesis and development of PD [37]. Therefore, oxidative stress modulating agents can effectively improve the clinical and behavioral symptoms of PD [38]. It is widely accepted that rotenone inhibits mitochondrial respiratory chain complex I, leading to the ROS production and oxidative stress [39]. Rotenone-induced oxidative stress is one of the suggested mechanisms for motor dysfunction in PD through the loss of dopaminergic neurons and dopamine depletion in the striatum [40, 41].

In the present study, daily administration of rotenone at the dose of 2 mg/kg for 5 weeks elicited a progressive reduction of body weight. In addition, rotenone administration significantly impaired motor activities which was confirmed by decreased latency time on the rotarod and shortened stride length. These results are consistent with previous studies showing that rotenone is used to induce animal PD models by causing pathological symptoms and motor deficits [42, 43]. Animal weight loss has also been shown in some rotenone-induced PD models, which may be due to the reduced food intake and gastrointestinal disorders [44, 45]. Whereas, dose-based administration of anethole significantly increased the body weight during the treatment period, which is probably due to the antioxidant effects of anethole on the gastrointestinal tract and improving digestion and absorption. In this regard, the protective effects of anethole on the intestine through antioxidant and anti-inflammatory mechanisms have been reported in several studies [46, 47].

The results of behavioral tests also indicated that anethole treatment significantly improved locomotor activity and motor coordination in rotenone-induced PD rats. This marked improvement in locomotion and coordination may reflect the protective effect of anethole against rotenone-induced damage in the substatia nigra (SN) dopaminergic neurons. Because in another study, we showed that treatment with anethole improves the motor symptoms in PD animals by increasing striatal dopamine levels and also reducing the death of striatal neurons [23]. Studies have also determined that anethole dithiolethione (derived from anethole) can be considered as a neuroprotective agent in PD due to its potential antioxidant effects against oxidative damage [48]. Another study has demonstrated that Fennel and its main constituent, Trans-anethole, have neuroprotective effect through their antioxidant properties in rats subjected to social isolation stress [49]. Therefore, the effects of anethole on improving motor activity in our study may also be due to its antioxidant property.

In this study, injection of rotenone significantly increased brain water content as well as BBB permeability, as demonstrated by Evans blue leakage. It is well known that oxidative stress is a major factor involved in the BBB disruption in neurodegenerative diseases [50]. Free radicals attack the cellular membranes, leading to damage intercellular tight junctions between the central nervous system (CNS) endothelial cells [51]. In agreement with these studies, our findings indicated that rotenone administration resulted in elevated level of MDA (a marker of lipid peroxidation), and decreased activity of SOD (the major antioxidant enzyme) in the striatum of rats. This suggests that in rotenone treated rats BBB impaired through the oxidative stress induced by overproduction of ROS. There is also growing evidence that dysfunction of the BBB is closely linked to increased CNS inflammatory response in numerous neurologic disorders such as PD [52, 53]. Therefore, oxidative stress and BBB dysfunction are extremely important in the etiology and progression of neurodegenerative diseases [54].

Our results showed that anethole significantly reduced Evans blue leakage as well as brain water content in rotenone-treated rats, indicating that anethole could prevent BBB impairment. Results also showed that anethole administration decreased MDA levels and enhanced SOD activity in the rat striatum. These data suggest that anethole may have a protective effect against BBB disruption by inhibiting rotenone-induced oxidative stress. The anti-inflammatory and antioxidant effects of anethole have also been demonstrated in previous studies [55–57]. For example, anethole dithiolethione has been shown to increase the cellular level of glutathione in cultured astrocytes of rat striatum [19]. Because astrocytes play an important role in protecting neurons against oxidative stress [58], this increase in antioxidant levels by anethole may help neuronal survival and maintain physiological function of CNS. Therefore, anethole seems to improve rotenone-induced neuronal damage and motor dysfunction by increasing antioxidant capacity and reducing neuroinflammation.

Electrophysiological results in the current study demonstrated that injection of rotenone markedly decreased the neuronal firing frequency. However, treatment with anethole at a high dose significantly increased the firing rate of striatal neurons compared to rotenone treated group. In previous studies, it has been suggested that rotenone-induced changes in the firing rate of striatal neurons occur following dysregulation of intracellular Ca^2+^ homeostasis through the plasmalemmal and mitochondrial Na^+^-Ca^2+^ exchanger (NCX) [59, 60]. It has also been demonstrated that rotenone induces α-syn aggregation in the substantia nigra neurons by increasing intracellular Ca^2+^ levels [61]. In addition, α-syn increases cytosolic Ca^2+^ levels by creating plasma membrane pores that facilitate the entry of extracellular calcium into the cytosol [62]. As a result, rotenone reduces spontaneous discharge of neurons through dysregulation of Ca^2+^ homeostasis, suggesting that rotenone-induced movement disorders in PD may be due to the altered neuronal firing in striatal circuits. Our results indicated that anethole could counteract the damaging effects of rotenone on the neuronal spontaneous activity of striatal circuits, but the precise mechanisms are yet to be established.

The results of our western blot analyses showed that administration of rotenone notably increased expression of α-syn and MAO-B in the striatum of rats, while the expression of α-syn and MAO-B were decreased in the group receiving rotenone and L-dopa. α-syn is a presynaptic neuronal protein and the major constituent of Lewy bodies, whose misfolding and aggregation in neurons is a prominent pathological hallmark of PD [63]. In accordance with our findings, animal model studies have shown that chronic systemic administration of rotenone by different routes facilitates the accumulation of α-syn and reproduce the neuropathological and behavioural symptoms of PD [64–66]. While levodopa treatment reduces the progression of PD by inhibiting the accumulation of α-syn in dopaminergic neurons [67]. MAO-B is an enzyme responsible for breakdown of dopamine and is involved in the production of free radicals by dopamine metabolism [68]. Rotenone has also been shown to increase MAO-B activity [69, 70], which is associated with increased ROS production, oxidative damage, and dopaminergic cell death [71, 72]. Because α‐Syn binds to MAO‐B and induces its enzymatic activity [73], The effect of levodopa on MAO-B activity is probably indirectly by inhibiting α-syn aggregation in neurons [67]. On the other hand, our results showed that expression of α-syn and MAO-B were decreased in the striatum of anethole treated rats. These findings were consistent with an in vitro study that showed that anethole dithiolethione reduced MAO-B activity in cellular extracts of cultured striatal astrocytes [24]. The effect of Anethole on MAO-B inhibition may also be due to its molecular structure or antioxidant properties. Although the underlying mechanisms that reduce α-syn expression by anethole have not yet been completely elucidated, it has been shown that the process of α-syn agreggation is inhibited by antioxidant compounds [74]. Moreover, it has been demonstrated that the inhibition of MAO enzymatic activity decreases the formation of α-syn fibrils [75, 76]. Therefore, anethole may reduce α-syn expression by inhibiting MAO-B or its antioxidant property.

## 5. Conclusion

Our results indicated that administration of anethole improved the motor performance and electrical activity of striatal neurons in rats treated with rotenone. In addition, we have shown that anethole alleviated oxidative stress and decreased the levels of α-syn and MAO-B activity as the two main pathological hallmark of PD. Taken together, these findings suggest that anethole can act as antioxidant and neuroprotective agent against rotenone rat model of PD.

## Supporting information

S1 Raw images Raw data files of all western blots from figure data. https://doi.org/10.7910/DVN/OAFMX0.
(PDF)

S1 Data (ZIP)
