# Supplementary material for: Anethole attenuates motor dysfunctions, striatal neuronal activity deficiency and blood brain barrier permeability by decreasing striatal α-synuclein and oxidative stress in rotenone-induced Parkinson’s disease of male rats
Source: PLoS One. 2023 Nov 16;18(11):e0294612. doi: 10.1371/journal.pone.0294612 (PMC10653401; doi:10.1371/journal.pone.0294612)
Supplement: S1 Raw images — https://doi.org/10.7910/DVN/OAFMX0. (PDF) [file pone.0294612.s001.pdf]

S1 Fig.

CONT	P.D	A250	L Dopa	CONT	P.D	A250	L Dopa

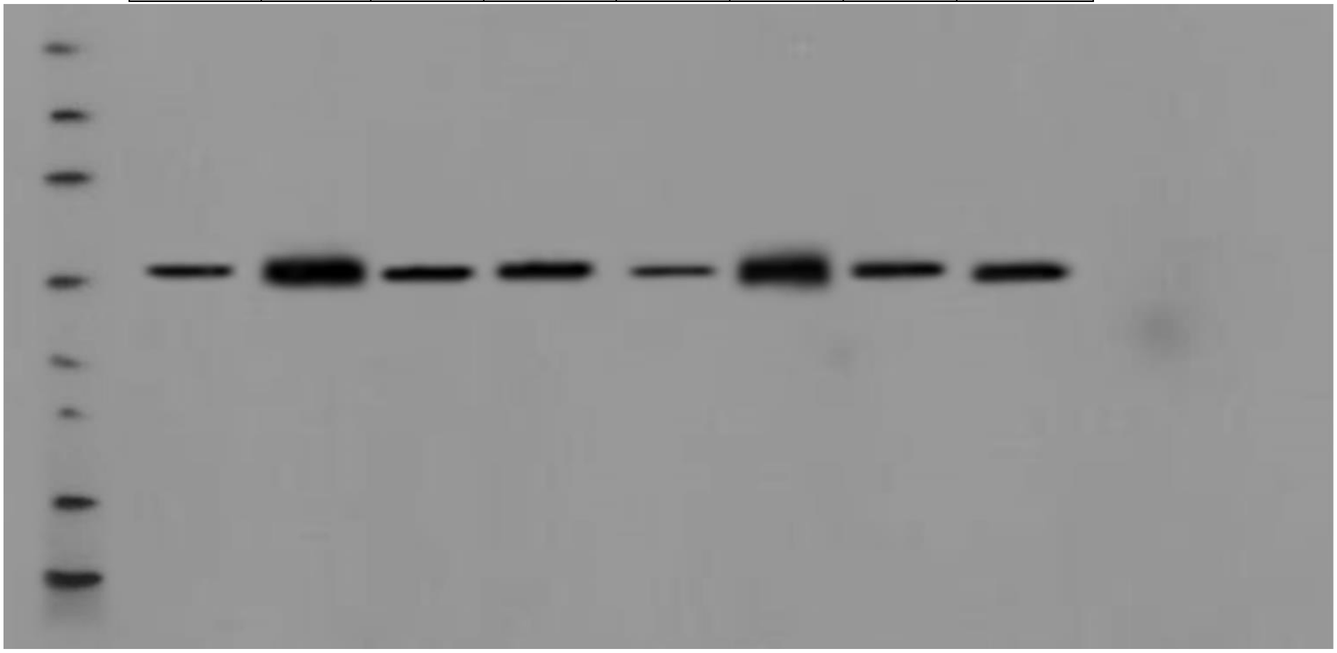

MAO-B, Run1

CONT	P.D	A250	L Dopa	CONT	P.D	A250	L Dopa

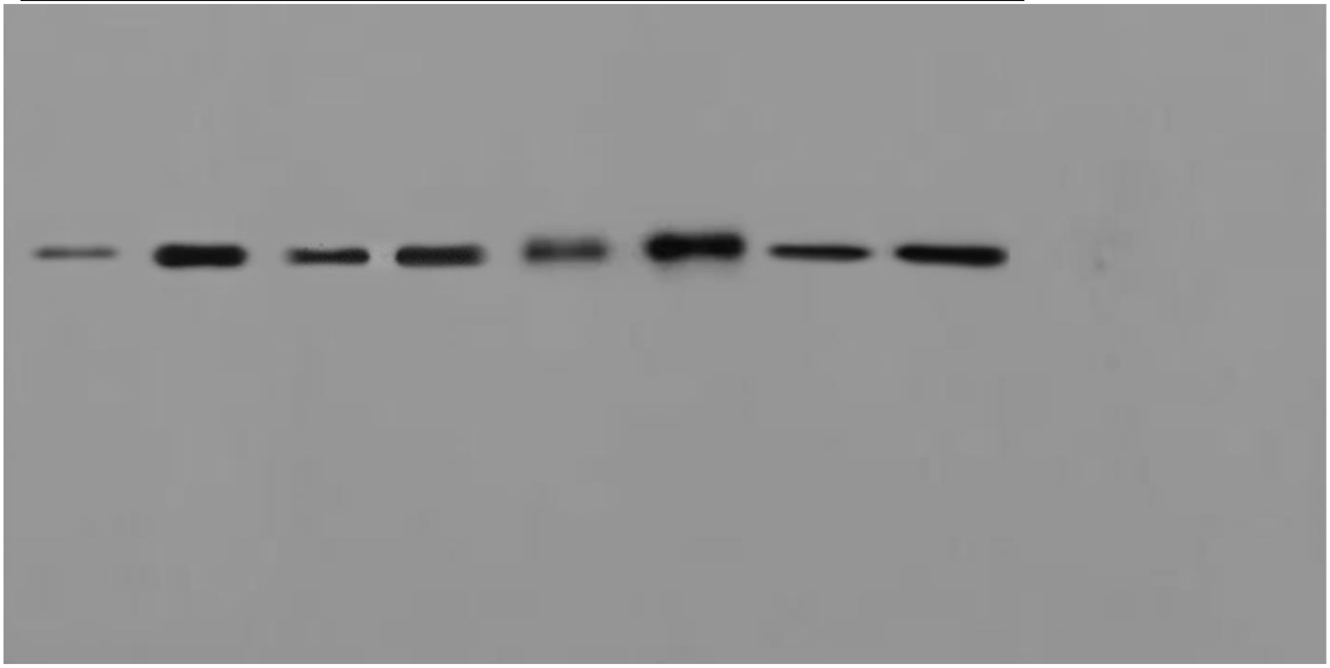

MAO-B, Run2

CONT	P.D	A250	L Dopa

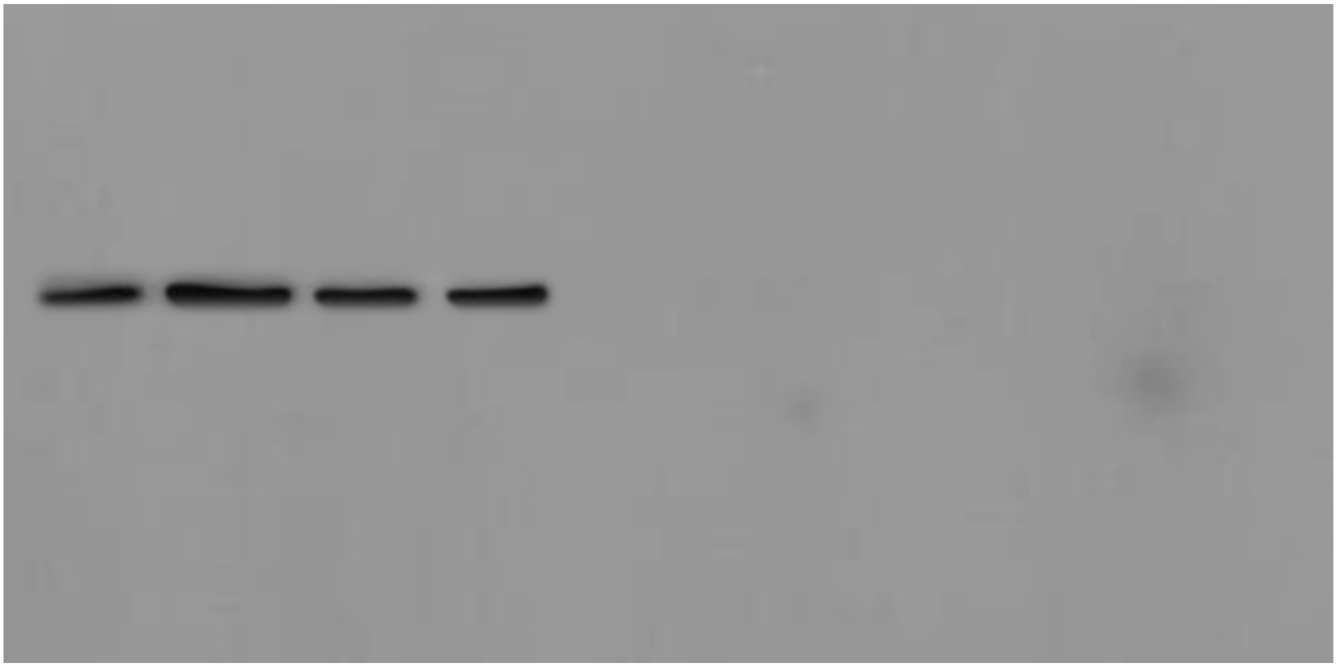

MAO-B, Run3

CONT	P.D	A250	L Dopa	CONT	P.D	A250	L Dopa

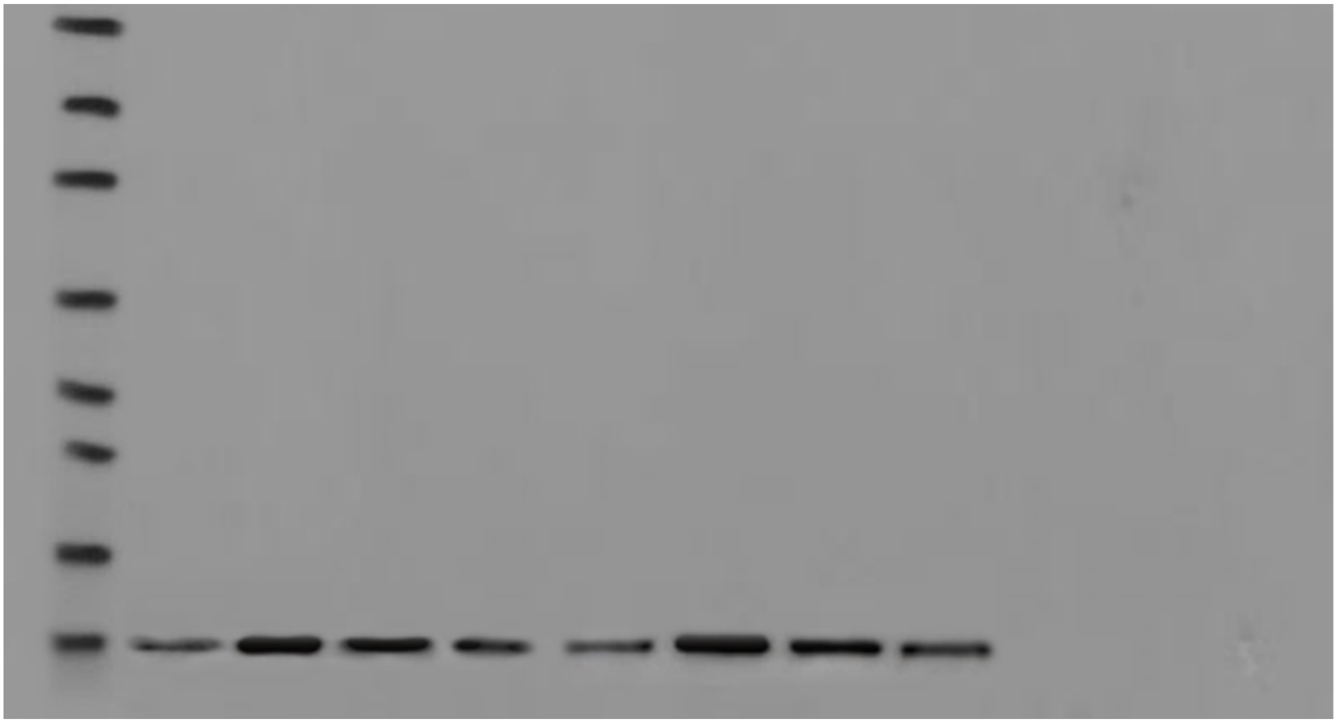

α-syn, Run1

CONT	P.D	A250	L Dopa	CONT	P.D	A250	L Dopa

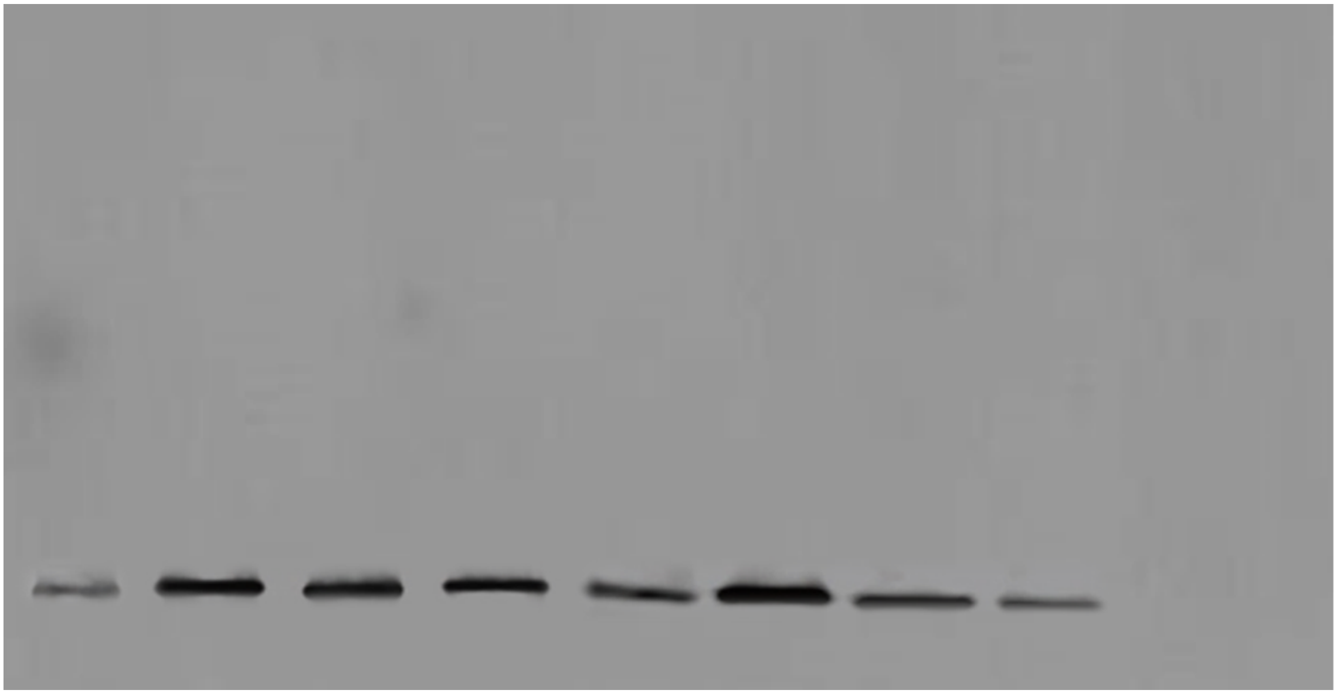

α-syn, Run2

CONT	P.D	A250	L Dopa

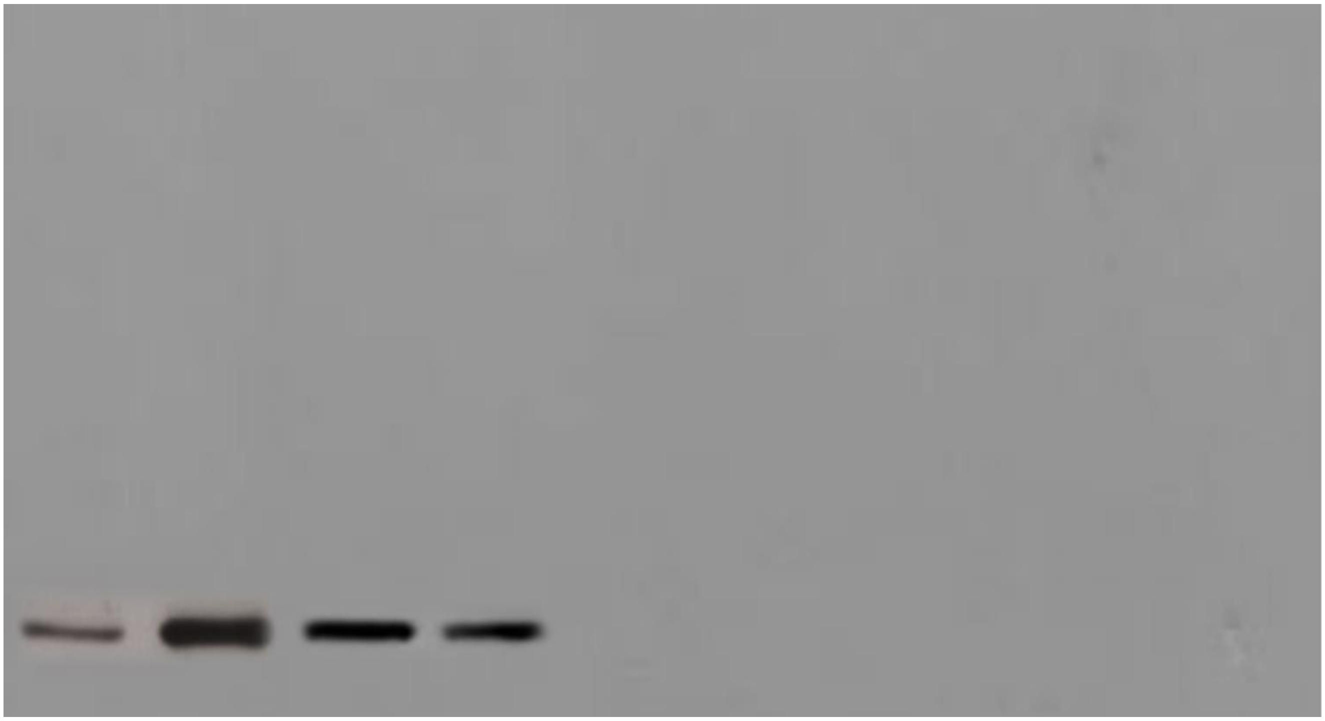

α-syn, Run

CONT	P.D	A250	L Dopa	CONT	P.D	A250	L Dopa

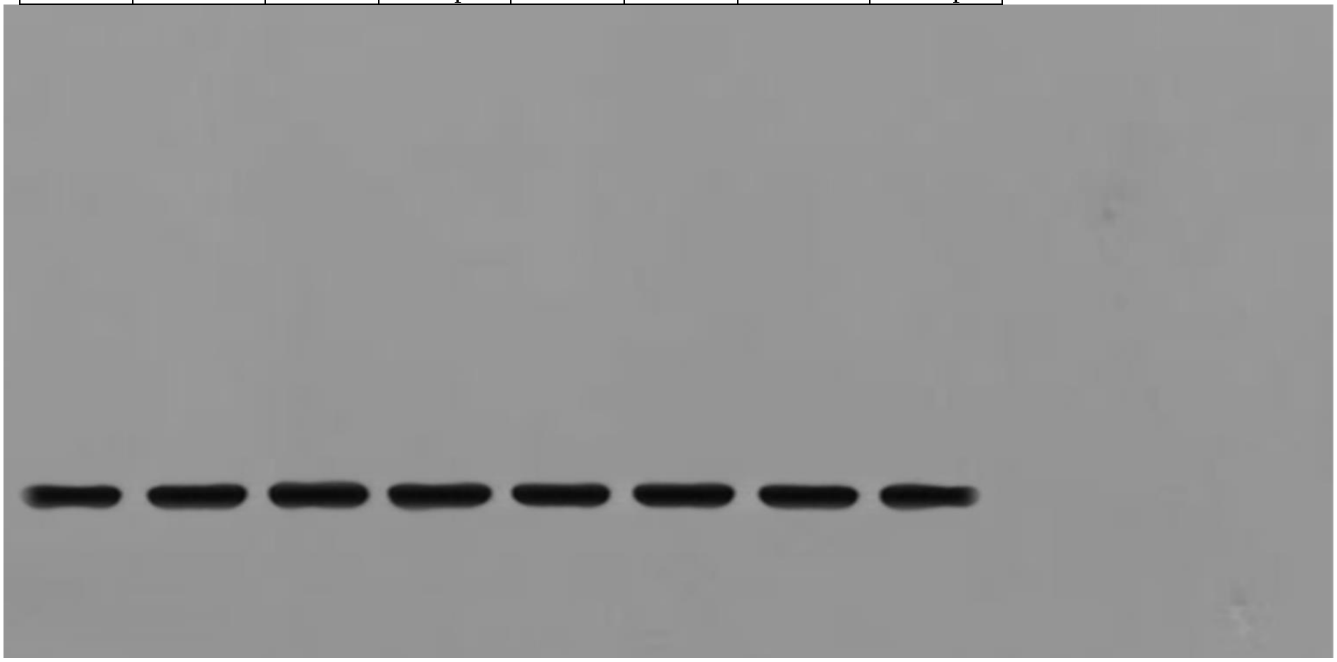

GAPDH, Run1

CONT	P.D	A250	L Dopa

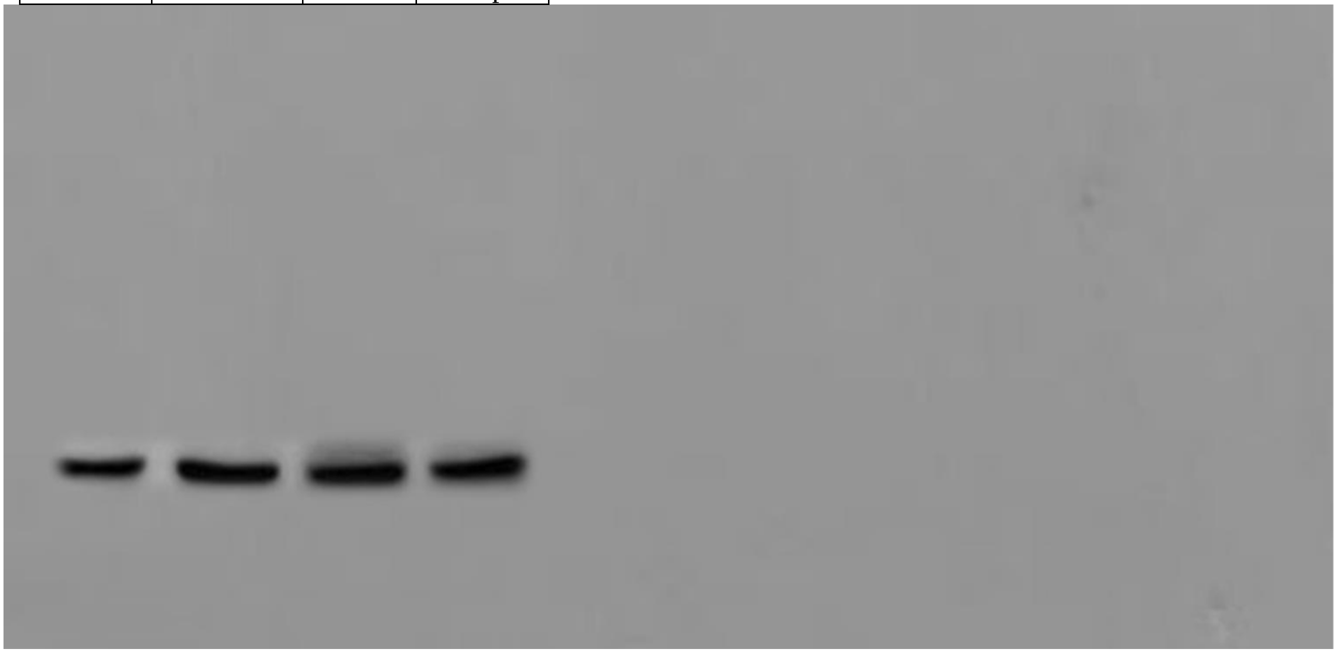

GAPDH, Run2
